# Sub-national landscape on the years of life lost due to COVID-19 pandemic in the major cities of Southern Philippines

**DOI:** 10.1093/pubmed/fdaf047

**Published:** 2025-05-07

**Authors:** Zython Paul Lachica

**Affiliations:** St. Cross College, 61 St Giles', Oxford OX1 3LZ, UK; Mindanao Center for Disease Watch and Analytics, Maguindanaoan Rd, College of Science and Mathematics Building, University of the Philippines Mindanao, Davao City 8000, Philippines

**Keywords:** COVID-19, disease burden, LMIC, sub-national, years of life lost

## Abstract

**Background:**

In Southern Philippines, 5 697 COVID-19 deaths were reported in the major cities from March 2020 to June 2022. The pandemic’s impact, despite the relatively modest death toll, was examined by analysing the Years of Life Lost (YLL) derived from disease surveillance datasets.

**Methods:**

The individual YLLs were calculated using the global disease burden approach applying 7% discounting rate and adjusting for sex, age-at-death, and the individual’s city-location. The YLLs were then aggregated on a monthly basis. Descriptive analytics were used to characterise the dynamic nature over time of the YLLs.

**Results:**

A total of 51 749.07 YLLs due to COVID-19 were estimated, i.e. 9.09 YLLs per death or 969.49 YLLs per 100 000 population. The monthly YLL ranged from 68.55 to 7 641.12. On average, the age-at-death is 59.68 years with males having younger age-at-death than females. Notably, the weekly COVID-19 incidences and deaths in Southern Philippines showed synchronous peaks.

**Conclusion:**

The average age-at-death in Southern Philippines is at least 10 years younger than both international age-at-death estimates and the Philippine life expectancy. The synchronous weekly peaks may highlight distinct pandemic dynamics for a low- and middle-income country. The YLL due to COVID-19 in Southern Philippines, at a sub-national level, is comparable to some country-level estimates, highlighting the impact of the pandemic on this island region alone.

## Introduction

The COVID-19 pandemic impacted nearly every country, leaving no doubt about its global reach, thus becoming a global concern. Most countries imposed wide-scale lockdowns to contain the virus. These measures were gradually eased as vaccines became available. Whilst the measures adopted were similar, the consequences of the pandemic still varied among countries. A country’s ability to mitigate the rising fatalities alongside other pandemic consequences (e.g. sharp economic downturns, escalating mental health challenges) is a good indicator of its resilience to health threats.^1^ Rather than resilience, the COVID-19 pandemic has magnified a country’s vulnerability to disease outbreak and its tendency to experience its worst-case consequences.

To fairly compare the impact of COVID-19 with other diseases across geographical territories, considering age and sex differences, it is essential to adopt a global health outcome measure that accounts for mortality disparities. Premature mortality, as a global health phenomenon, has important implications for health policy,^2^ especially in the pursuit of universal health care. For instance, a country’s health benefit package can be redesigned to prioritise treating diseases that lead to massive premature deaths.^3^ In turn, healthcare resources can be targeted where they are most needed instantaneously. On the other hand, premature death has repercussions to a country’s economy. A premature death represents missed opportunities across sectors, resulting in decreased tax revenue and a potential reduction in the production of goods, services, or knowledge, which are crucial to the functioning and growth of the economy. Essentially, premature death brought by the pandemic can be thought as an irreversible societal damage. It is irreversible due to the loss of life it entails, and its societal impact arises from the ripple effects that extend beyond the individual death, affecting families and communities.

The Years of Life Lost (YLL) is a metric measuring premature mortalities that accounts both frequency of deaths and the age at which these deaths happened. It is an important metric as it quantifies how much life has been cut short for the population due to a disease.^4^ In the face of a new pandemic threat, the profiled YLL from a previous or other pandemic provides a critical baseline for pandemic preparedness planning. Health agencies and their technical working groups can use this baseline measure when evaluating the effectiveness of any proposed control measures. Interventions that can prevent the most YLL at a low cost are deemed as effective in controlling disease spread. Such an evaluation is useful in identifying the most suitable control interventions before they are implemented.

The Philippines is one of the countries to have the longest series of wide-scale lockdown and amongst the Asian countries to have the highest fatalities. Understanding the impact of COVID-19 solely at the national level would be regressive for an archipelagic country; having a sub-national focus is needed to promote inclusivity for health-related policy matters. It is worth mentioning that Southern Philippines hosts a significant portion of the country’s regional administrative offices, including an autonomous region, major cities for commerce and tourism, and is renowned for its high biodiversity. Administratively, Southern Philippines operates within the framework of policies set at the national capital, ensuring alignment with national governance.^5^

The scientific papers on COVID-19 in the Philippines have covered several dimensions of the pandemic including its transmission dynamics, impact on public health and education, and potential mitigation strategies.^6^ Many studies have also focused on specific geographical sites as well. In Southern Philippines, several papers have examined the (i) landscape of COVID-19 infection among patients,^7–8^ (ii) logistical implementation and optimal levels of control interventions,^9–12^ and (iii) predicting the spread of the disease through various modelling approaches.^13–14^ Most of these studies focus on disease incidence and death toll as health outcomes. Few papers, such as those by Migriño and Bernardo-Lazaro,^15^ have studied disease burden of COVID-19 at a country-level, with limited focus on Southern Philippines. Thus, this paper aims to provide a sub-national estimate of the impact of COVID-19 in Southern Philippines by analysing the YLL at the height of the pandemic.

## Methodology

### Study site, data sources, and ethics statement

The YLLs were derived based on COVID-19 mortality data from major cities in Southern Philippines (refer to Table 1). These major cities were selected in consideration of COVID-19’s urban-centric nature that tends to significantly affect densely populated areas with bustling economies.^16–17^

**Table 1:** Study sites per region and their geographic characteristics.

Region	City	Relative contribution of the city’s COVID-19 cumulative cases^d^
Zamboanga Peninsula	City of Pagadian^a^	9%
	Zamboanga City^b,c^	48%
Northern Mindanao	Cagayan de Oro City^a,b,c^	27%
Davao	Davao City^a,b,c^	58%
SOCCSKSARGEN	City of Koronadal^a^	10%
	General Santos City^b^	25%
Caraga	Butuan City^a,b^	25%
Bangsamoro Autonomous Region in Muslim Mindanao	Cotabato City^a,b^	26%

Note: ^a^Regional city centre, ^b^Densest component local government unit (LGU) in the region, ^c^Independent chartered cities; ^d^City cumulative case count/Regional cumulative case count.

The COVID-19 patient line lists used in this study were obtained through partnering with the following regional Department of Health—Centers for Health Development (DOH—CHDs): Zamboanga Peninsula (IX), Northern Mindanao (X), and Davao (XI), as well as with the Integrated Provincial Health Office of South Cotabato in SOCCSKSARGEN (XII). The line lists were reviewed and verified by their respective Regional and Provincial Epidemiology and Surveillance Units (RESUs and PESUs), as reported through their official communications. For other major cities where verified data from regional health centres were unavailable at the time of data collection, line lists from the COVID-19 DOH Data Drop^18^ were used. As a caution, the data obtained from DOH Data Drop were supplied by the DOH-accredited test laboratories and may not necessarily have been reviewed by the regional or provincial surveillance units.

The line lists include an anonymised patient code, sex, age, location, and/or dates of birth and death. This set of information were used to calculate for the YLL from the individual level. Information on the health status upon admission were also recorded. The author adhered to the Department of Health Joint Memorandum Circular No. 2020–0002 for processing and disclosing COVID-19-related data in the Philippines. The protocol was reviewed by the Davao Center for Health Development—Joint Research Ethics Committee with protocol number JREC-202315.

The linelist covered disease surveillance starting 15 March 2020 or the date when most of the Southern Philippine cities enforced a wide-scale lockdown. The last surveillance date included is 30 June 2022 or the last day prior to the assumption into office of the Philippine government officials elected in 2022. The cut-off date was selected to avoid any discussion bias coming from the change of government administration.

Computation for the GBD-based YLL

The YLL was computed following the global burden of disease approach (GBD), wherein time discounting factor is used.^19^ The discrete-time-discounted GBD YLL for the *i*th COVID-19 death at the *j*th city was computed using Eq. (1).^20^


(1) \begin{equation*} GBB\ YL{L}_{ij}=\sum_{t=0}^T\frac{1}{{\left(1+r\right)}^t} \end{equation*}

where $T$ is the undiscounted YLL and $r$ is the discounting rate at 7% following the Philippine Health Technology Assessment guideline.^21^ The value of $T$ for COVID-19 deaths less than 60 years old and more than 60 years old was computed accordingly as detailed below.

For individuals whose age-at-death is less than 60 years old, $T$ is obtained by getting the difference between their *life expectancy at birth* (*LEB*) and *age-at-death* (*AD*). The *LEB* is determined by the individual’s location and sex which can be found in a Philippine Statistics Authority report.^22^ On the other hand, the *active life expectancies* (*ALE*) were used to compute for $T$ for individuals whose *AD* is at least 60 years old. Following the *Philippine ALE* estimates of Cruz and colleagues,^23^ the base age is first determined, either 60, 70, or 80 years, and the corresponding *ALE* is then applied. Individuals between the ages of 60 and 69 have a base age of 60, and the same rule applies for subsequent age groups. Due to the unavailability of regional *ALE* estimates at the time of writing, *Philippine ALE* estimates were used to derive *ALEs* for major cities in Southern Philippines, referred to as *Southern Philippines-adjusted ALE* (ALE*), as shown in Eq. (2)


(2) \begin{equation*} {ALE}_{ij}^{\ast }=\frac{\left( Philppine\ ALE\right)\left( LE{B}_j\right)}{Philippine\ LE} \end{equation*}

where $Philppine\ LE$ is the nationally published life expectancy at birth by the National Statistics Office.^22^  Table 2 summarizes the Southern Philippines-adjusted active life expectancies per age group.

**Table 2:** Southern Philippines-adjusted active life expectancies per age group used in this paper.

Location	Sex	Southern Philippines-adjusted Active Life Expectancy		
		[60,70) y.o.	[70,80) y.o.	>80 y.o.
National level	Male	16.80	10.90	6.30
	Female	22.20	14.70	8.50
Region IX	Male	16.61	10.78	6.23
	Female	21.83	14.46	8.36
Region X	Male	16.67	10.82	6.25
	Female	21.84	14.46	8.36
Region XI	Male	16.70	10.84	6.26
	Female	21.86	14.48	8.37
Region XII	Male	16.65	10.80	6.24
	Female	21.85	14.47	8.37
Region XIII	Male	16.46	10.68	6.17
	Female	21.62	14.31	8.28
BARMM	Male	15.82	10.26	5.93
	Female	20.75	13.74	7.94

Subsequently, ${ALE}_{ij}^{\ast }$ is then added to the $base\ ag{e}_i$ before subtracting the recorded AD. Lastly, it is conservatively assumed that *YLL* = 1 whenever ${T}_{ij}$ becomes less than or equal to zero (e.g. a woman passing away at the age of 89). Eq. (3) summarizes the computation of $T$.


(3) \begin{equation*} {T}_{ij}=\left\{\begin{array}{ll} LEB_{ij}-A{D}_i,& A{D}_i<60\kern0.33em \mathrm{and}\kern0.33em {T}_{ij}>0,\\{}\left( base\kern0.33em ag{e}_i+ AL{E}_{ij}^{\ast}\right)-A{D}_i,& A{D}_i\ge 60\kern0.33em \mathrm{and}\kern0.33em {T}_{ij}>0,\\{}1,& \mathrm{otherwise}.\end{array}\right. \end{equation*}

As a caveat, the life expectancies at birth in Southern Philippines (average LEBs at 69.51 years for males and 75.11 years for females) is slightly lower than that of other island groups and the national average (71.26 years for males and 77.54 years for females),^22^ which likely accounts for the lower ALEs observed in Table 2. Furthermore, it is assumed that the mortality patterns across all age groups, as well as for those aged 60 and above, show little to no difference between the considered regions and the national level.

### Descriptive analytics

This study considered the temporal dimension of COVID-19 YLL, instead of a single-point estimate, to capture dynamic nature of YLLs. Descriptive analytics focuses on understanding historical events by exploring what has happened. Data visualisation techniques were used to present these events effectively.^24^ To provide an overview of the extent of the COVID-19 impact, the following temporal datasets pooled from the major cities in Southern Philippines were visualised in this paper:

- COVID-19 cases according to severity and deaths;
- COVID-19 YLL;
- proportion of health status upon admission; and
- distribution of YLL and the age-at-death.

The temporal datasets were overlaid with wide-scale community quarantines and alert level systems, vaccine rollouts, and the establishment of testing laboratories. This approach facilitated the visual representation of COVID-19 patterns alongside these interventions. All visualisations were created using *R* software.^25^

## Results

### COVID-19 timeline in the major cities of Southern Philippines

**Figure 1:**
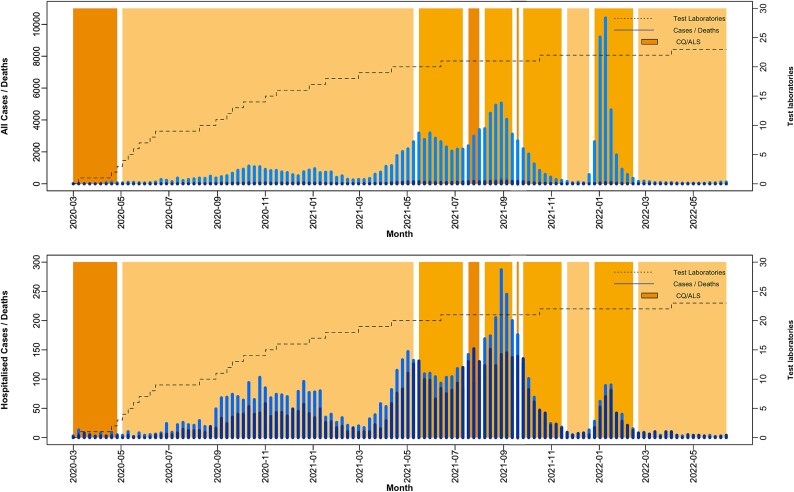
The COVID-19 timeline in the major cities of southern Philippines overlaid with the implementation of the CQ, ALS, and the number of test laboratories. Darker shades correspond to stricter CQ/ALS. The plotted CQ/ALS was based on the widely implemented CQ/ALS every week by the different cities. The earliest implementation of the ALS amongst the major cities occurred during the first week of November 2021. The upper panel displays the overall incidence and deaths, whilst the lower panel presents a zoomed-in view, focusing on hospitalised incidence (i.e. severe and critical COVID-19 cases) and deaths.

The COVID-19 incidence and deaths, Community Quarantine (CQ) and the Alert Level System (ALS) timeline, and the number of test laboratories established in Southern Philippines is shown in Fig. 1. The CQ has four levels, with level 4 being the most stringent (enhanced community quarantine) and Level 1 being the most relaxed (modified general community quarantined). The CQ Level 4 is characterised with strict home quarantine, transportation suspension, and regulated provision of food and essential healthcare services.^6^ Permissive economic activities are allowed with CQ Level 1, and individuals are urged to adhere to the required minimum health standards.^13^ Meanwhile, the ALS is modified version of the CQ system but focuses on targeted restrictions and allows economic activities outside the lockdown areas.^6^

Periods of prolonged increases in COVID-19 cases or hospitalisations are often followed by the implementation of stricter CQ or ALS measures. For example, CQ was raised to Level 3 in late May to mid-July 2021 after a steady rise in hospitalisations reported from late March to mid-May 2021. A similar pattern was also observed when CQ was escalated to its strictest level from early to late August 2021, following a sustained increase in cases and hospitalisations from early June to mid-August 2021. In late August 2021, CQ was downgraded to its second-strictest level. However, with hospitalisations peaking and consistently high case counts and deaths from late August to late September 2021, CQ returned to its strictest level in early October 2021. Subsequently, a significant decline in COVID-19 cases and deaths was noted, leading to a relaxation of CQ/ALS measures. Additionally, ALS was tightened to its strictest level during the final COVID-19 surge in Southern Philippines, which recorded the highest case counts. Over the course of the pandemic, the number of COVID-19 testing facilities increased from zero to 23.

### Years of life lost timeline in the major cities of Southern Philippines

The 5 697 individual deaths attributed to COVID-19 during the period considered in this study accounted for a total of 51 749.07 YLLs, which translates to approximately 9.0863 YLLs per death. The lowest recorded monthly YLL was 68.55 in June 2020 and the highest was 7 641.12 in September 2021. Regarding demographics, the average age-at-death in the major cities of Southern Philippines was 59.68 years, with individuals aged 48 years and older representing 80% of total deaths. The early adult and working-age group contributed 53.49% of the deaths, followed by the elderly population at 43.75% and the young population at 2.76%. Moreover, 55.35% of the deaths were recorded among men, who had a slightly lower average age-at-death (58.97 years) compared to women (60.57 years).

**Figure 2:**
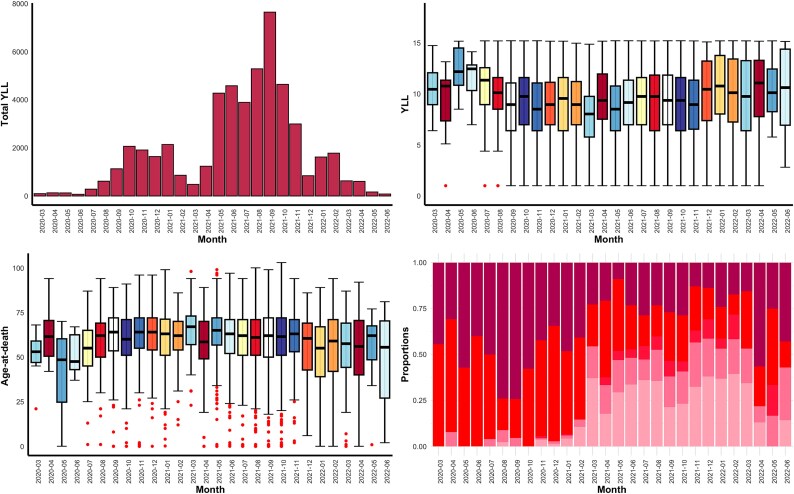
Overview of the pooled COVID-19 YLL from the major cities in Southern Philippines from March 2020 to June 2022. Top-left to bottom-right: (a) monthly total COVID-19 YLL, (b) boxplot of the monthly COVID-19 YLL, (c) boxplot of the age-at-death, and (d) monthly composition of the COVID-19 cases according to patient health status upon admission. For the monthly proportions only five out of eight cities have patient admission status information and dark colours represent more severe admission statuses, whilst lighter colours indicate less severe statuses. Admission statuses considered are asymptomatic, mild to moderate, severe, and critical. Significant events: (1) the start of vaccination rollout in March 2021 and (2) the detection of the Delta and Omicron variants in July and December 2021, respectively.

Figure 2 (top-left panel) shows relatively minimal YLL during the period when intra- and inter-city travel restrictions were in place (March to June 2020). YLL steadily increased from July 2020 to January 2021, followed by a brief decline. A sharp rise in YLL is observed around a month after the vaccination campaign began, maintaining elevated levels until peaking in September 2021, after which YLL gradually decreased. A shift in the age-at-death distribution is also noted during vaccine deployment, with the average age-at-death moving from 63.57 years to 60.97 years. In the top-right and bottom-left panels, an increase in COVID-19-related deaths among younger individuals is seen starting from July 2020, coinciding with a period of more relaxed interventions. Meanwhile, the bottom-right panel shows a rise in the proportion of reported asymptomatic cases during the period when more COVID-19 testing laboratories became operational.

## Discussion

### Main finding of the study

The COVID-19 case counts and death toll in Southern Philippines exhibit synchronous weekly peaks. Further, the computed age-at-death in Southern Philippines is at least 10 years younger than international estimates^4^^,^^26–27^ and the country’s life expectancy. Lastly, the aggregated COVID-19 YLL from the major cities in Southern Philippines is comparable to some country-level YLL estimates.

### What is already known on this topic

Synchronous temporal disease peaks have been observed in countries with the highest case-fatality ratios, such as Mexico^28^ and Peru.^29^ Meanwhile, a 2- to 3-week delay between the peak in disease incidence and fatalities is common in countries like the United States and Germany.^30–31^

In terms of demographic characteristics of COVID-19 fatalities, younger deaths were observed in low- to middle-income and middle-income countries like Brazil, Bangladesh, and India.^32–34^ The Philippines on the other hand has a relatively younger population^35^ and younger susceptible group. At the population level, lifting mobility restrictions increases the pool of susceptible individuals, providing the virus with more potential hosts for infection. Additionally, many low-to-middle income countries report a high proportion of workers in informal employment, with limited access to health and related services.^36^

When comparing to international estimates, the YLL per 1 000 population due to COVID-19 in Southern Philippines is estimated to be 4.88, notably higher than in South Korea and Norway (between 2020 and 2021).^37^ In Chile, the YLL per 100 000 population is 1 247.1,^38^ whilst in the major cities of Southern Philippines, it is estimated to be 969.49 (between 2020 and 2022).

### What this study adds

The results of this study suggest that these synchronous peaks may highlight distinct pandemic dynamics in low- and middle-income countries. Additionally, the younger population and the large informal workforce may contribute to the observed younger age-at-death.^39^ Furthermore, this paper compares YLL-disease burden estimates for the Southern Philippine region, which is comprised of six administrative regions, against other countries, highlighting COVID-19’s substantial impact on this island region alone.

Quantifying location-specific YLL provides a crucial baseline for comparing the impact of COVID-19 with other diseases and potential future pandemics in Southern Philippines. This approach ensures that local health burdens are not overshadowed by national estimates. YLL profiling captures the dynamic nature of disease burden, offering insights into how it shifts even within short time spans. Such analysis can inform the design of more nuanced policies and interventions, as opposed to relying solely on national-level data. Additionally, YLL serves as a measure of irreversible societal impacts, such as premature deaths, helping in evaluating population-level health resilience or the effectiveness of control interventions.

The implementation of CQ/ALS reflects the broader need to adapt to shifting COVID-19 conditions without relying on the strictest tier or level unnecessarily.^40^ The 9-week period of strict measures after the outbreak’s onset may be considered reasonable, given the absence of vaccines and the limited testing infrastructure at the time. However, variations in protocols, such as isolation procedures, and logistical challenges across cities affected the CQ/ALS implementation. These complexities present potential difficulties in achieving the intended outcomes.^41^ Furthermore, assessing the cost-effectiveness of these interventions is crucial to inform future pandemic responses. Profiling these interventions based on the COVID-19 experience will ensure that previous learnings are not overlooked. Whilst such assessments may be challenging, a dynamic evaluation of cost-effectiveness, particularly in the context of a new pandemic, is essential. This approach allows for a more strategic layering of control measures, which can be guided by the profiled YLL estimates.^1^

### Limitations of the study

Whilst these deaths are tagged as COVID-19 deaths, some may not be solely attributable to the virus and instead to underlying comorbidities. However, due to the lack of access to relevant data, caution is advised when interpreting the estimated YLLs presented here. The most appropriate interpretation is that these are the YLLs observed during the COVID-19 pandemic.

It is common practice to express disease burden as the sum of YLLs and YLDs (years lost due to disability), i.e. disability-adjusted life year or DALYs. However, the disability component of DALY is often subject to criticism, particularly regarding how disability is defined.^42^ Moreover, in the context of COVID-19, infected individuals may remain asymptomatic and continue to function normally. Importantly, when comparing the duration of illness (YLD) to the YLL, the YLD becomes negligible. Thus, this analysis focuses on YLL.

## Notes

**Zython Paul Lachica**, BS, MSc
